# 3′-hydroxy-3,4,5,4′-tetramethoxystilbene, the metabolite of resveratrol analogue DMU-212, inhibits ovarian cancer cell growth *in vitro* and in a mice xenograft model

**DOI:** 10.1038/srep32627

**Published:** 2016-09-02

**Authors:** Hanna Piotrowska-Kempisty, Marcin Ruciński, Sylwia Borys, Małgorzata Kucińska, Mariusz Kaczmarek, Piotr Zawierucha, Marcin Wierzchowski, Dawid Łażewski, Marek Murias, Jadwiga Jodynis-Liebert

**Affiliations:** 1 Department of Toxicology, Poznan University of Medical Sciences, Dojazd 30 St., PL-60-631 Poznan, Poland; 2 Department of Histology and Embryology, Poznan University of Medical Sciences, Swiecickiego 6 St. PL-60-781 Poznan, Poland; 3 Department of Anatomy, Poznan University of Medical Sciences, Swiecickiego 6 St., PL-61-781 Poznan, Poland; 4 Department of Clinical Immunology, Poznan University of Medical Sciences, Rokietnicka 5d St., PL-60-806 Poznan, Poland; 5 Department of Chemical Technology of Drugs, Poznan University of Medical Sciences, Grunwaldzka 6 St., PL-60-780 Poznan, Poland

## Abstract

In screening studies, the cytotoxic activity of four metabolites of resveratrol analogue 3,4,5,4′-tetramethoxystilbene (DMU-212) against A-2780 and SKOV-3 ovarian cancer cells was investigated. The most active metabolite, 3′-hydroxy-3,4,5,4′-tetramethoxystilbene (DMU-214), was chosen for further studies. The cytotoxicity of DMU-214 was shown to be higher than that of the parent compound, DMU-212, in both cell lines tested. Since DMU-212 was supposed to undergo metabolic activation through its conversion to DMU-214, an attempt was made to elucidate the mechanism of its anti-proliferative activity. We found that in SKOV-3 cells lacking p53, DMU-214 induced receptor-mediated apoptosis. In A-2780 cell line with expression of wild-type p53, DMU-214 modulated the expression pattern of p53-target genes driving intrinsic and extrinsic apoptosis pathways, as well as DNA repair and damage prevention. Regardless of the up-regulation of p48, p53R2, sestrins and Gaad45 genes involved in cancer cell DNA repair, we demonstrated the stronger anti-proliferative and pro-apoptotic effects of DMU-214 in A-2780 cells when compared to those in SKOV-3. Hence we verified DMU-214 activity in the xenograft model using SCID mice injected with A-2780 cells. The strong anti-proliferative activity of DMU-214 in the *in vivo* model allowed to suggest the tested compound as a potential therapeutic in ovarian cancer treatment.

Resveratrol (3,4′,5-*trans*-trihydroxystilbene) has widely been studied for its anti-bacterial, anti-fungal, anti-inflammatory, anti-oxidative and anti-cancer effects^1^,^2^. Although the pleiotropic activities of resveratrol have been revealed, its therapeutic potential is limited due to low bioavailability and rapid elimination from the body^3^. Therefore, structural modifications of the resveratrol scaffold have been conducted to produce synthetic derivatives with improved pharmacokinetic parameters. The substitution of hydroxyl (-OH) groups of resveratrol for methoxy groups (-OMe) may increase molecule stability, thus making it less susceptible to phase II conjugation reactions *in vivo*^4^. The analysis of the structure-activity relationships showed that the introduction of additional methoxy groups into the stilbene backbone potentiated the cytotoxic effects of the compound. Furthermore, methylated resveratrol analogues bearing -OMe groups at positions 3,5- and 3,4,5- of the trihydroxystilbene moiety have been reported to demonstrate stronger pro-apoptotic activity when compared to other stilbene derivatives^5^. One such molecule, DMU-212 (3,4,5,4′-tetramethoxystilbene), has been revealed to have anti-cancer activity via cell cycle arrest and activation of apoptosis^6^,^7^,^8^,^9^,^10^,^11^,^12^.

DMU-212 has been shown to evoke receptor and mitochondrial apoptotic pathways in transformed fibroblasts, breast, liver, colon and ovarian cancer cell lines^6^,^8^,^9^,^10^,^11^. However, a few studies have suggested that the metabolic products of DMU-212 might be responsible for the anti-proliferative effects of the parent compound^6^,^13^,^14^,^15^.

On the basis of our own and the findings of other authors, we synthesized four metabolites of DMU-212, i.e. 3′-hydroxy-3,4,5,4′-tetramethoxystilbene (DMU-214), 4′-hydroxy-3,4,5-trimethoxystilbene (DMU-281), 4-hydroxy-3,5,4′-trimethoxystilbene (DMU-291) and 3-hydroxy-4,5,4′-trimethoxystilbene (DMU-807), and examined them in A-2780 and SKOV-3 ovarian cancer cell lines in order to identify the compound showing the highest cytotoxic effects. As a result of the screening analysis, DMU-214 was chosen for further investigation.

The aim of the present study was to evaluate the molecular mechanism of the potential anti-proliferative activity of DMU-214 in A-2780 and SKOV-3 ovarian cancer cell lines. The activation of apoptosis was assayed by the following analyses: RNA microarrays, the expression of 35 apoptosis-related proteins, cell cycle modulation and activation of caspases- 8, -9 and -3/7. Since the A-2780 cell line was found to be more sensitive to DMU-214 than SKOV-3, the anti-tumour properties of the compound tested *in vivo* were investigated using SCID mice injected with A-2780 cells.

## Results

### Effect of the metabolites of DMU-212 on the proliferation of A-2780 and SKOV-3 cells

**Figure 1:**
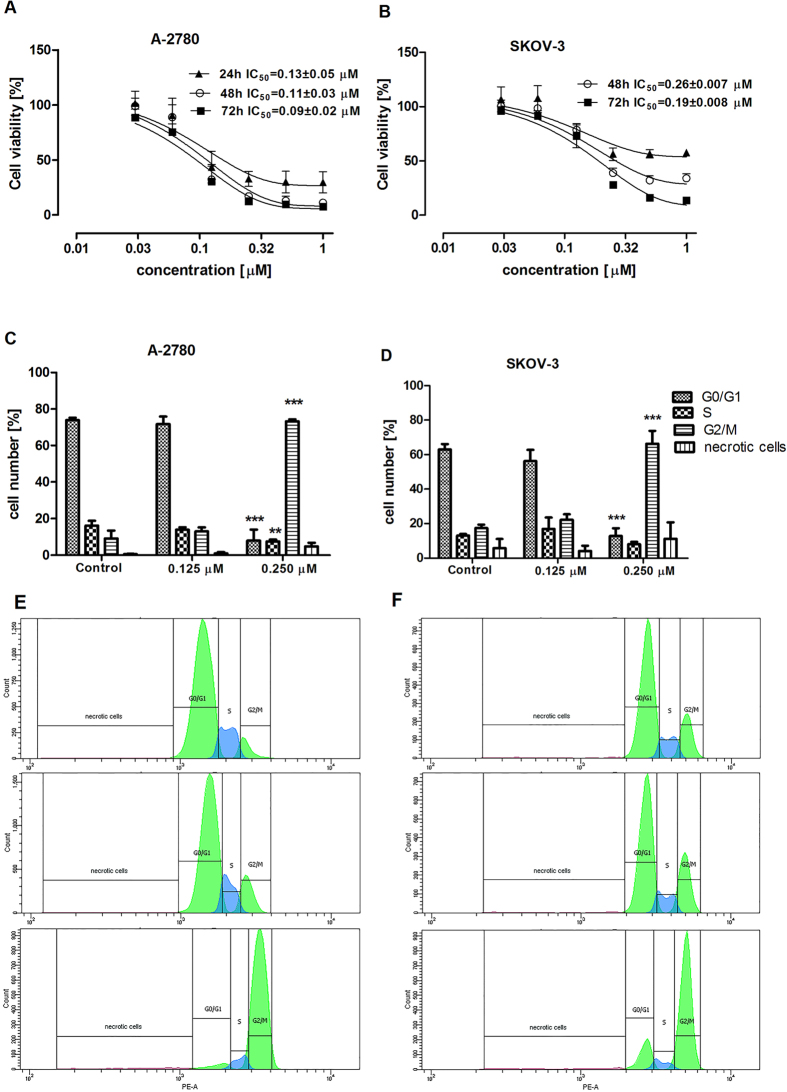
Effect of DMU-214 on A-2780 and SKOV-3 cell proliferation. After 24 h, 48 h and 72 h of treatment with DMU-214 in the range of concentration 0–1 μM, IC_50_ values were determined by MTT assay in the (**A**) A-2780 and (**B**) SKOV-3 cell line. Cell cycle phase distribution was analysed by flow cytometry in (**C**) A-2780 and (**D**) SKOV-3 cells treated for 24 h with a vehicle or with 0.125 μM and 0.250 μM of DMU-214. Representative histogram from cell cycle analysis in the (**E**) A-2780 and (**F**) SKOV-3 cell lines after DMU-214 treatment for 24 h. Results of three independent replicates are presented as mean ± SD. ***P < 0.001 and **P < 0.01 indicate a significant difference from the control.

The inhibitory effect of four metabolites tested against the A-2780 and SKOV-3 cell lines was evaluated after 24 h, 48 h and 72 h at a concentration of 10 μM by MTT assay (Table 1). From among the four metabolites of DMU-212, DMU-214 showed the highest cytotoxic activity against the tested cell lines. The cytotoxicity of the selected compound, DMU-214, was first assessed in a concentration range of 0-10 μM after 24 h, 48 h and 72 h, respectively. The lowest concentration tested (1 μM) caused a sudden reduction of the viability of A-2780 and SKOV-3 cells (data not shown). Hence the anti-proliferative effects of DMU-214 were examined in the concentration range of 0–1 μM. The IC_50_ values for A-2780 cells were 0.13 ± 0.05 μM (24 h), 0.11 ± 0.03 μM (48 h) and 0.09 ± 0.02 μM (72 h), and for the SKOV-3 cell line they were 0.26 ± 0.007 μM (48 h) and 0.19 ± 0.008 μM (72 h), (Fig. 1A,B). In order to assess which phase of the cell cycle was affected, the A-2780 and SKOV-3 cell lines were treated with 0.125 μM and 0.250 μM of DMU-214 for 24 h, and the percentage of cells in each phase of the cell cycle was determined by flow cytometry. It was demonstrated that A-2780 and SKOV-3 cells were arrested with the higher concentration of DMU-214 (0.250 μM) in the G2/M phase by 590% and 380%, respectively as compared to control (Fig. 1C,D). The exposure of A-2780 cells to 0.250 μM of DMU-214 also resulted in a decreased number of cells in G0/G1 and S phase as compared to untreated controls. Similarly, the number of SKOV-3 cells treated with the higher concentration was found to be reduced in G0/G1 and S phase, however, the latter was not statistically significant. The number of necrotic cells in both cell lines tested was also assayed; no statistically significant differences as compared to the controls were noted. Figure 1E,F show the representative histograms from flow cytometry analysis in A-2780 and SKOV-3 cell lines treated with 0.125 μM and 0.250 μM of DMU-214.

### Effect of DMU-214 on apoptosis in A-2780 and SKOV-3 cell lines

**Figure 2:**
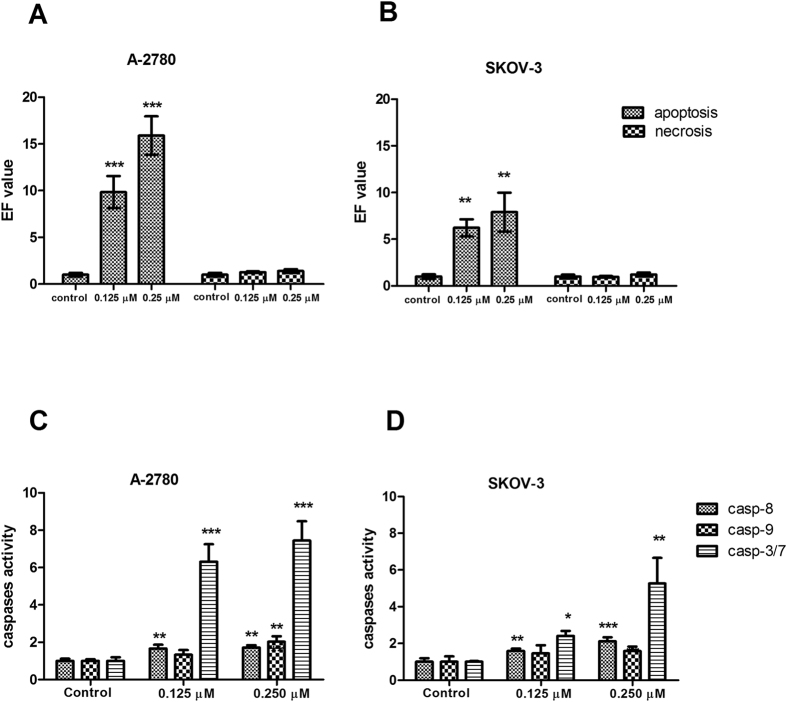
Effect of DMU-214 on apoptosis. A-2780 and SKOV-3 cells were treated for 24 hours with a vehicle or with 0.125 μM and 0.250 μM of DMU-214. Apoptosis and necrosis induction was assayed by the ELISA test and expressed as an enrichment factor (EF) in the (**A**) A-2780 and (**B**) SKOV-3 cell lines. Casp-8, -9 and -3/7 activities were determined in the (**C**) A-2780 and (**D**) SKOV-3 cell lines. Results of three independent replicates are presented as mean ± SD. ***P < 0.001, **P < 0.01 and *P < 0.05 indicate a significant difference from the control.

The induction of apoptosis in A-2780 and SKOV-3 cells treated with 0.125 μM and 0.250 μM of DMU-214 for 24 h was assayed by the Cell Death Detection ELISA^PLUS^ test. The pro-apoptotic activity of DMU-214 was expressed as an enrichment factor (EF) (Fig. 2A,B). Both concentrations that were tested caused an increase in the nucleosomes level in A-2780 lysates, EF = 9.84 ± 1.72 and EF = 15.89 ± 2.05, respectively. The statistically significant pro-apoptotic effect of DMU-214 at the tested concentrations was also found in the SKOV-3 cell line (EF = 6.22 ± 0.92 and EF = 7.91 ± 2.08), however, to a lesser extent than in A-2780. The number of necrotic cells in the A-2780 and SKOV-3 supernatants was also evaluated; no statistically significant differences as compared to the controls were noted. Figure 2C shows that the activity of both initiator caspases -8 and -9 was increased in A-2780 cells treated with the higher concentration of DMU-214 for 24 h, 1.6- and 2.0-fold, respectively as compared to control. However, no effect of the compound tested at a concentration of 0.125 μM was observed in caspase-9 activation. The activity of effector caspases-3/7 was found to increase ~6.5- and 7.5-fold in A-2780 cells treated with both concentrations of DMU-214, respectively. In the SKOV-3 cell line, no changes in caspase-9 were observed, while the activity of caspase-8 was increased ~1.5- and 2.1-fold at concentrations of 0.125 μM and 0.250 μM of DMU-214, respectively (Fig. 2D). The activation of caspase-3/7 was also up-regulated 2.4- and 5.3-fold in the SKOV-3 cell line following treatment with both concentrations of DMU-214, respectively.

### Microarray analysis and Real Time quantitative PCR (RT-qPCR)

**Figure 3:**
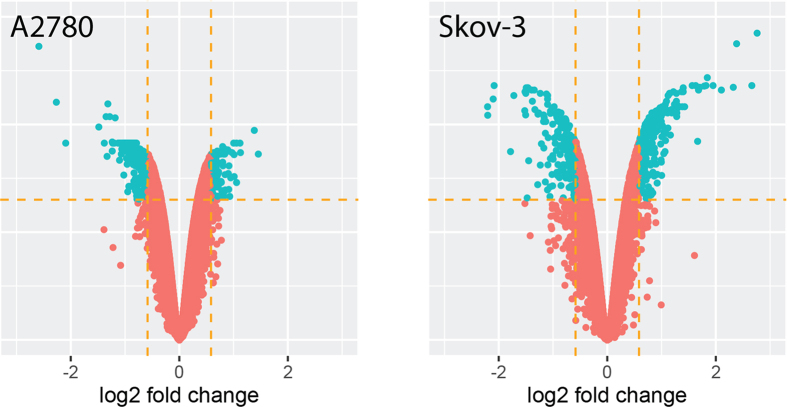
Affymetrix® Human Genome U219 microarray. Gene expression profile in A-2780 and SKOV-3 cells affected by DMU-214. A-2780 and SKOV-3 cells were incubated for 24 h with a vehicle or with 0.250 μM of DMU-214 and then analysed by means of Affymetrix® Human Genome U219 microarray. Genes whose fold changes were larger than the cut-off value (fold >1.5 and fold >−1.5 with p < 0.05) were considered as differentially expressed. A total of 765 and 838 genes met these selection criteria in the A-2780 and SKOV-3 cell lines, respectively. In A-2780 cells, 522 genes were up-regulated, whereas 243 were down-regulated in relation to the controls. In the SKOV-3 cell line, the expression of 366 genes was increased while that of 472 was decreased as compared to the control. Each dot presented on the graph corresponds to one transcript.

To obtain a complete view of the A-2780 and SKOV-3 gene expression profile affected by DMU-214, we performed whole gene expression analysis by Affymetrix^®^ Human Genome U219 microarray, which we used to examine the expression of more than 36 000 transcripts. The dynamic profile of whole gene expression, in A-2780 and SKOV-3 cells treated with the higher concentration of DMU-214 for 24 h, was shown in the volcano plot (Fig. 3). Each dot presented on the graph corresponds to one transcript. The genes for which the fold change was larger than the cut-off value (fold >1.5 and fold >−1.5 with p < 0.05) were considered as differentially expressed. In total, 765 and 838 genes met these selection criteria in the A-2780 and SKOV-3 cell lines, respectively. In A-2780 cells, 522 genes were up-regulated, whereas 243 were down-regulated in relation to the control group. In the SKOV-3 cell line, the expression of 366 genes was increased while that of 472 was decreased as compared to the control.

**Figure 4:**
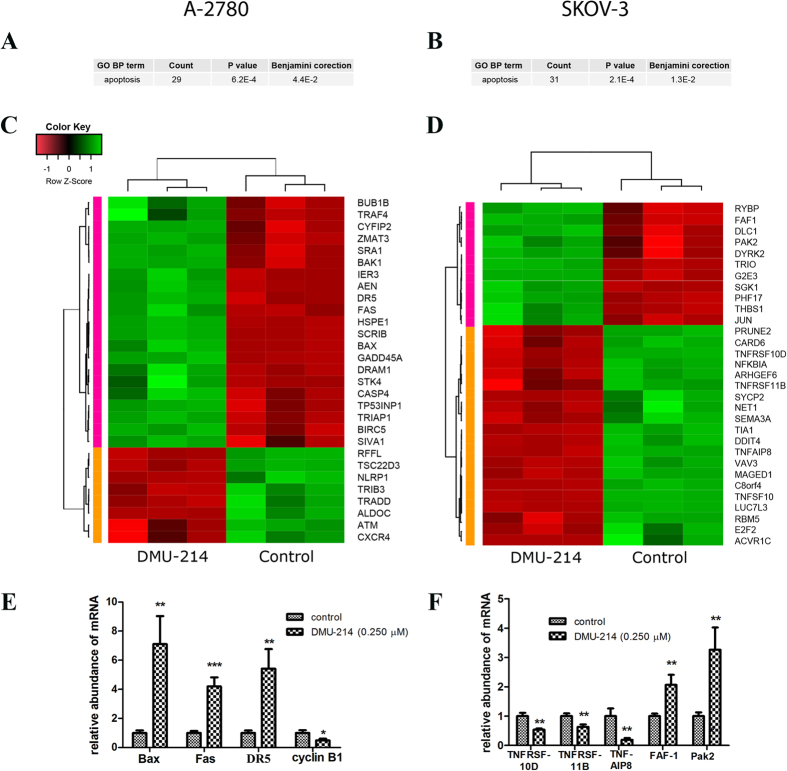
Heat map representation of microarray analysis in A-2780 and SKOV-3 cells treated with DMU-214. The set of differentially expressed apoptosis-related genes obtained by the Fisher exact test with Benjamini correction in (**A**) A-2780 and (**B**) SKOV-3 cell lines. Clusterization of apoptosis-related genes presented as a heat map in (**C**) A-2780 and (**D**) SKOV-3 cells. Signal intensity acquired from the microarray analysis is represented by colours; green colour–increase in expression, red colour–decrease in expression. RT-qPCR was used to validate the microarray assays. Relative abundance of (**E**) Bax, Fas, DR5 and cyclin B1 mRNAs in A-2780 cell line and (**F**) TNFRSF10D, TNFRSF11B, TNFAIP8, FAF-1 and Pak2 mRNAs in SKOV-3. The tested cell lines were treated for 24 h with a vehicle or DMU-214 (0.250 μM). Results of three independent replicates are presented as mean ± SD. ***P < 0.001, **P < 0.01 and *P < 0.05 compared to the control.

DAVID (Database for Annotation, Visualization and Integrated Discovery) software was used for extraction of the genes involved in apoptosis regulation. For this purpose, the whole set of differentially expressed genes was loaded into the DAVID search procedure. Using the Fisher Exact Test with Benjamini correction, we noticed that our set of differentially expressed genes was highly represented by the apoptosis-related genes in terms of the Gene Ontology biological function database (Fig. 4A,B). We found 29 and 31 differentially expressed genes involved in the apoptosis process in A-2780 and SKOV-3, respectively. This set of genes was subjected to a detailed analysis by hierarchical clusterization and presented as a heat map (Fig. 4C,D). In A-2780 cells, 21 genes were up-regulated, whereas eight genes were down-regulated as compared to the untreated cells. In the SKOV-3 cell line, the expression of 11 genes was increased while that of 20 was decreased in relation to the control group. The data obtained by means of the RNA microarray method were validated by RT-qPCR for selected genes. As is demonstrated in Fig. 4E, DMU-214 caused a marked increase in the expression of Bax, DR5 and Fas in the A-2780 cells 7-, 6- and 4- fold, respectively. Concurrently, DMU-214 suppressed the mRNA level of cyclin-B1 by 50% in the A-2780 cell line. Figure 4F shows that the tested compound decreased mRNA expression of TNFRSF10D and TNFRSF11B by ~50%, while the TNFAIP8 transcript level was reduced by 80% in the SKOV-3 cell line. Furthermore, DMU-214 caused an increase in FAF-1 and Pak2 mRNA expression ~2.0- and 3.0-fold, respectively.

**Figure 5:**
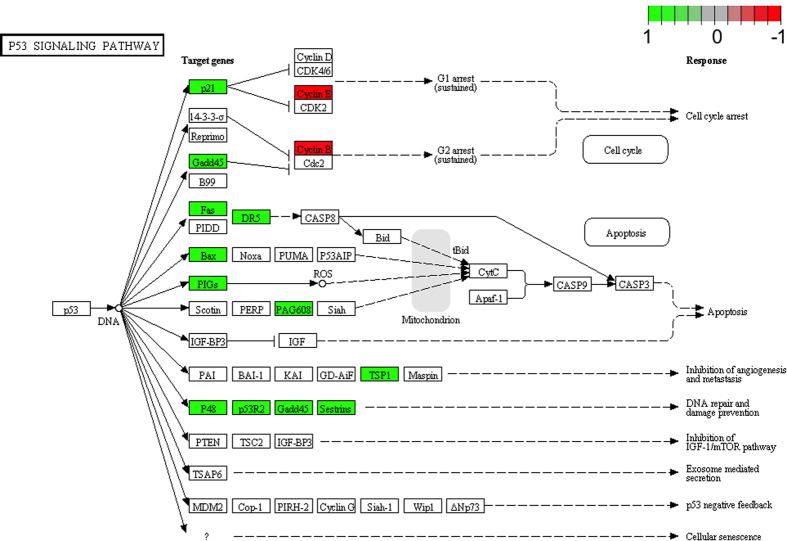
p53 signalling pathway in A-2780 cells treated with DMU-214. Expression changes of p53 target genes are mapped by colours; green colour–increase in expression, red colour–decrease in expression.

In A-2780, we performed an analysis of the p53 signalling pathway, in which differentially expressed genes were mapped by colours. The green colour represents up-regulated and the red colour represents down-regulated expression levels in relation to the control (Fig. 5).

### Protein expression analysis

**Figure 6:**
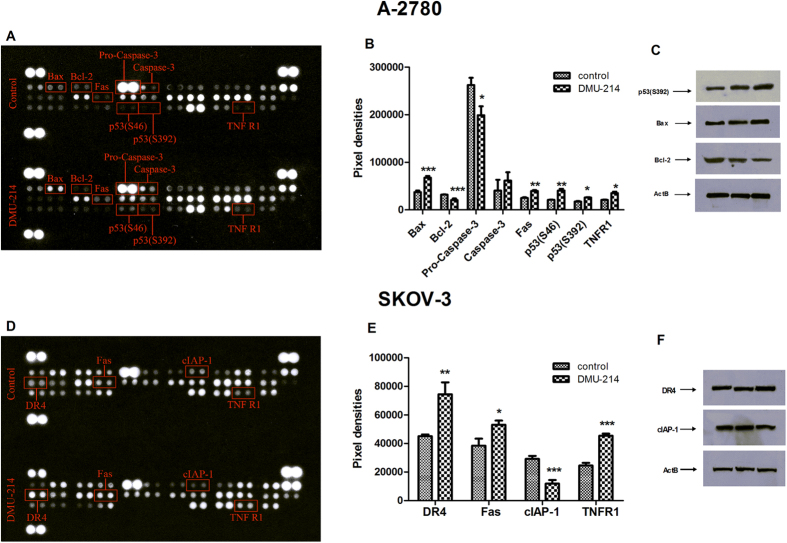
Effect of DMU-214 on apoptosis-related gene and protein expression in A-2780 and SKOV-3 cells. A representative picture of apoptosis array analysis. (**A**) Expression changes of two phosphorylated forms of p53 and other apoptosis-related proteins: Bax, Bcl-2, pro-caspase 3, casp-3, Fas and TNFR1 in A-2780 cells and (**D**) DR4, Fas, cIAP-1 and TNFR1 protein level changes in SKOV-3 cells are highlighted in red in the scan image. Densitometric studies were used to analyse the level of indicated proteins in (**B**) A-2780 and (**E**) SKOV-3 cells treated for 24 h with a vehicle or DMU-214 (0.250 μM). Results of three replicates are presented as mean ± SD.***P < 0.001, **P < 0.01 and *P < 0.05. Western blot was performed to validate apoptosis array analysis. **(C)** An increase in Bax and p53(S392) and a decrease in the Bcl-2 protein level in A-2780 cells and (**F**) increase in DR4 and decrease in cIAP-1 protein level in SKOV-3.

Proteome Profiler Human Apoptosis Array was performed to indicate changes in apoptosis-related protein expression triggered by DMU-214 in the A-2780 and SKOV-3 cell lines (Fig. 6A,B,D,E). The protein analysis showed that two phosphorylated forms of p53 and other six apoptosis-related proteins were changed in A-2780 cells treated with DMU-214 (0.250 μM) for 24 h. The tested compound caused an increase in the level of Bax, Fas, p53(S46), p53(S392), casp-3 and TNFR1 proteins from ~30% to 200%, which was paralleled to a decrease in Bcl-2 and pro-caspase-3 protein expression by ~35% (Fig. 6A,B). The DMU-214-induced changes in the p53(S392), Bax and Bcl-2 protein levels were confirmed by Western blot analysis (Fig. 6C). However, the protein expression changes were more pronounced as compared to those of the screening analysis. In the SKOV-3 cell line treated with DMU-214 (0.250 μM) for 24 h, the Proteome Profiler Human Apoptosis Array indicated an increase in the DR4 and TNFR1 protein level by ~100% as well as in Fas by ~30%, respectively. cIAP-1 protein expression was found to be down-regulated by 50%, as compared to the untreated controls (Fig. 6D,E). The DMU-214 induced changes in DR-4 and cIAP-1 proteins expression were proved by Western blot analysis (Fig. 6F). The changes in DR-4 and cIAP-1 proteins level corresponded to those of screening analysis.

### Anti-tumour activity of DMU-214 in a xenograft model *in vivo*

**Figure 7:**
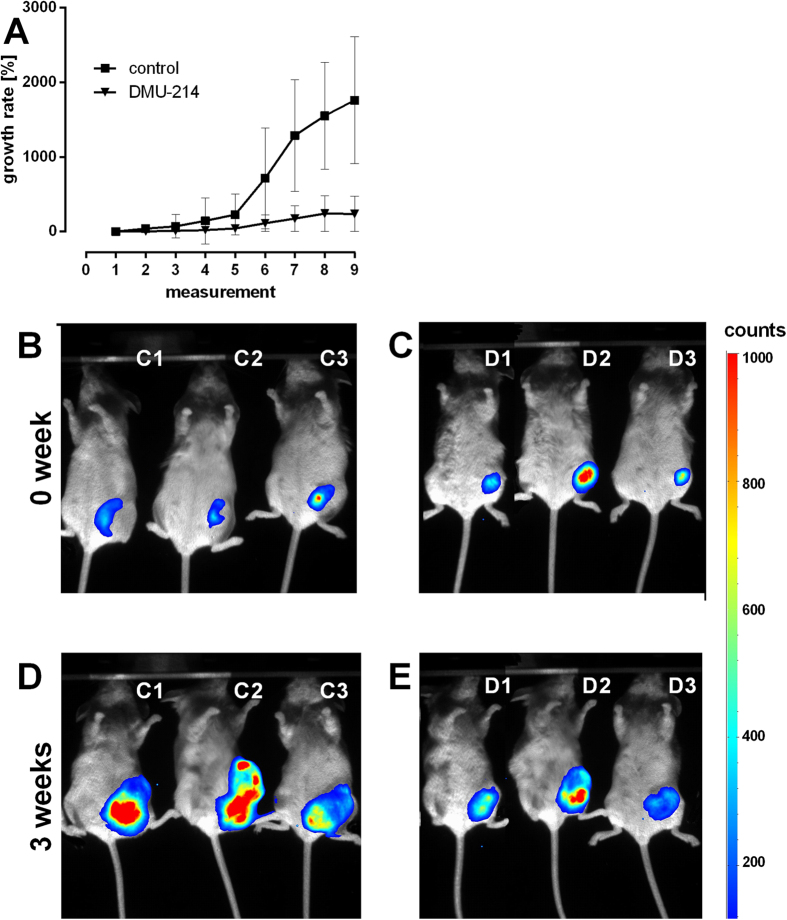
Effect of DMU-214 on the tumour burden in SCID mice with A-2780 ovarian cancer xenografts. Changes in tumour development in mice treated with DMU-214 (40 mg/kg b.w.) for 3 weeks. Representative control mice are marked as C1, C2 and C3, while the DMU-214-treated group is marked with D1-D3. Panels of the figure show: (**A**) the percentage of tumour growth during the whole time period of the experiment (9 measurements). (**B**) images of the control mice–week 0, **(C)** images of DMU-214-treated mice–week 0, (**D**) images of the control mice–week 3, (**E**) images of DMU-214-treated mice–week 3.

The potential anti-tumour effect of DMU-214 *in vivo* was evaluated in human ovarian cancer xenografts in mice. At day 3 after injection of A-2780 cells, mice with a similar level of bioluminescence were divided into two groups (control and DMU-214-treated). The mice were treated with DMU-214 at 40 mg/kg three times a week for the entire period of observation, and the results are shown in Fig. 7. The tested compound caused significant suppression of tumour growth as compared to the control (Fig. 7A). Figure 7B–E display the images of decreased tumour-derived luminescence in 3 representative mice treated with DMU-214 for three weeks. DMU-214 at a dose of 40 mg/kg b.w. was well tolerated and did not cause any adverse effects. There were no statistically significant changes in body weight between control and DMU-214-treated mice.

## Discussion

We evaluated the cytotoxicity of four metabolites of DMU-212 in A-2780 and SKOV-3 human ovarian cancer cell lines. In the screening analysis, the most cytotoxic metabolite, DMU-214, was selected for further studies. Androutsopoulos *et al*. also reported that DMU-214 exerted the strongest anti-proliferative activity among the other metabolites of DMU-212 in breast and liver cancer cells, however, they observed that the cytotoxic effects of DMU-214 were similar, and even slightly lower than those of DMU-212^6^. On the contrary, we showed that the cytotoxicity of DMU-214 was significantly higher than that of DMU-212 in A-2780 and SKOV-3 ovarian cancer cells. Therefore, it can be suggested that DMU-212 undergoes metabolic activation through its conversion to DMU-214 in A-2780 and SKOV-3 ovarian cancer cells, thus we attempted to elucidate the mechanism of the anti-proliferative activity of DMU-214 in the ovarian cancer model.

The cytotoxicity of the parent compound, DMU-212, has been revealed to correlate with its ability to induce apoptosis in liver, colon, breast and ovarian cancer cells^6^,^9^,^10^,^13^,^14^,^15^. Similarly, Androutsopoulos *et al*. reported that the anti-proliferative activity of DMU-214, the metabolite of DMU-212, was related to the induction of apoptosis in liver and breast cancer cell lines^6^. In the present study we confirmed this observation, since the strong cytotoxic effects of DMU-214 were observed on A-2780 and SKOV-3 ovarian cancer cells in which the tested compound induced apoptosis, as evidenced by the high EF value. However, the SKOV-3 cell line was noted to be less sensitive to DMU-214 than A-2780, since the IC_50_ and EF values differed significantly between both of the tested cell lines. The SKOV-3 cell line has been revealed to be resistant to many chemotherapeutics used routinely in anti-cancer therapy, e.g. it has been shown that the anti-proliferative activity of the common cytostatic agent cisplatin was significantly higher in A-2780 than in the SKOV-3 cell line^16^,^17^. Several studies demonstrated that A-2780 cells express wild-type p53, while in SKOV-3 the p53 protein was not detected^18^,^19^. Hence it can be suggested that the different sensitivities of both ovarian cancer cell lines to potential anti-cancer agents, including DMU-214, might depend on the status of p53 expression.

In the A-2780 cells we identified, by means of transcriptome analysis, mRNA expression changes of 765 genes, including 29 apoptotic ones. Furthermore, our results pointed at the p53 pathway of apoptosis. We did not find significant differences in the p53 mRNA level between control and DMU214-treated cells. However, the up-regulation of two phosphorylated forms of the p53 protein (Ser 46 and 392) was revealed as a result of DMU-214 treatment. Since the phosphorylation of p53 regulates its ability to activate the expression of apoptotic target genes, we can suggest that 14 genes, identified by microarray analysis, were regulated in a p53-dependent manner in A-2780 cells. Some studies have indicated that the principal role of p53 is related to the mitochondrial pathway of apoptosis through the p53-Bax network^20^. DMU-214 has been reported to up-regulate p53 as well as the Bax/Bcl-2 ratio in liver and breast cancer cells^6^. We found that DMU-214 up-regulated Bax mRNA 7-fold in the A-2780 ovarian cancer cell line. We also noted that DMU-214 significantly increased Bax and decreased the Bcl-2 protein level. Since Bax/Bcl-2 ratio >1 is widely known to be associated with promotion of p53-dependent apoptosis, the obtained results seem to confirm the contribution of p53 in apoptosis. Furthermore, we showed up-regulation of other p53-induced genes, PAG608 and PIGs, which have been linked to the mitochondrial pathway of apoptosis.

It is well known that caspase-9 is an initiator of the caspase cascade in the mitochondrial death pathway. Once activated, it cleaves and activates downstream effector caspases, including casp-3 and -7^21^. In this study, DMU-214 was found to increase initiator casp-9 and executive casp-3/7 activity in A-2780 ovarian cancer cells. Moreover, protein analysis revealed that DMU-214 caused down-regulation of procaspase-3 and up-regulation of the casp-3 protein level. Since casp-9 has been revealed to promote cleavage of procaspase-3 to casp-3, leading to mitochondrial apoptosis, our results indicate the contribution of this apoptotic response to DMU-214 in A-2780 cells. However, casp-3 is known to be activated via both mitochondria- and receptor- mediated pathways of apoptosis^22^. We noted that DMU-214 caused a slight increase in the activity of casp-8, which is a key enzyme in the extrinsic pathway of apoptosis. Extracellular apoptosis is mediated by death receptors, mainly Fas, TNFR-1, DR-3, -4 and -5^23^. We showed that DMU-214 up-regulated Fas and DR5 mRNA levels over 5-fold in A-2780 ovarian cancer cells. In addition, proteome analysis revealed an increased Fas and TNFR1 protein level after DMU-214 treatment. Since these two receptors activate casp-8, it might be suggested that receptor-mediated apoptosis was also involved in the cytotoxicity of DMU-214 in A-2780 cells. These results are consistent with our previous findings that the parent compound DMU-212 regulated the expression of genes specific to the extracellular mechanism of apoptosis in ovarian cancer cells^10^. Accordingly, in the SKOV-3 cell line lacking p53 we found, by means of transcriptome analysis, changes in the expression pattern of 31 apoptosis-related genes, including 8 extrinsic ones. Furthermore, protein analysis showed that DMU-214 caused an increase in the protein levels of death receptors Fas, TNFR1 and DR4, which are members of the TNF family. TNFAIP8 has been revealed to suppress TNF-mediated apoptosis by inhibiting casp-8 activity^24^. In the present study, we demonstrated that DMU-214 decreased the mRNA level of TNFAIP8, accompanied by up-regulated activity of casp-8 in SKOV-3 cells. These results suggest that the tested compound has induced extrinsic pathway of apoptosis since the activation of casp-8 has been in general associated with receptor-mediated apoptosis. Simultaneously, DMU-214 has been shown to decrease other anti-apoptotic TNFRSF10D and TNFRSF11B genes belonging to the TNF family. Several studies indicated that FAF-1 binds to the Fas ligand and potentiates Fas-induced apoptosis^25^. We found a significant increase in mRNA expression of FAF-1 as well as Pak2, which is activated by caspase-3 in Fas-mediated apoptosis. Furthermore, DMU-214 has been shown to increase the activity of casp-3/7 without any changes in casp-9. In view of these results, we can suggest that DMU-214 triggers the extrinsic pathway of apoptosis in a Fas- and TNF-mediated manner in the SKOV-3 cell line. No changes in the expression of genes and proteins driving intrinsic apoptosis were observed. It is well known that induction of mitochondrial apoptosis is mainly regulated by the p53 protein^20^. Hence we can suggest that the inability of DMU-214 to induce the intracellular pathway of apoptosis in SKOV-3 cells may result from the lack of p53 expression in these cells.

DMU-214 has been found to induce G2/M cell cycle arrest accompanied by apoptosis in breast and liver cancer cells^6^. Our results are in agreement with Androutsopoulos *et al*.’s findings since we showed that DMU-214 triggered the G2/M block and apoptosis in SKOV-3 and A-2780 cell lines. Progression from the G2 to M phase is regulated predominantly by cyclin B1, which is in cross-talk with p53^26^,^27^. In the A-2780 cell line, DMU-214 was shown to decrease the cyclin B1 mRNA level, paralleled to the block in cell cycle progression in the G2/M phase leading to p53-dependent apoptosis. Our results correspond to other authors’ findings showing that cell cycle arrest in the G2/M phase was associated with the inhibition of cyclin B1^28^,^29^. Although we did not find any changes in cyclin B1 expression in SKOV-3, cell cycle arrest in the G2/M phase was observed. However, the number of cells arrested in the G2/M phase was significantly lower as compared to A-2780. Since p53 is not detected in SKOV-3 cells, we can suggest that they were blocked in the G2/M phase in a manner that was different than in A-2780 cells.

Currently, chemotherapy is a treatment option for most types of ovarian cancer^30^. However, the effectiveness of chemotherapeutics can be limited by multiple tumour cells’ defence mechanisms^31^. We found that in the A-2780 cell line, DMU-214 caused increased expression of p53 target genes p48, p53R2, Gaad45 and sestrins, whose transcripts are implicated in DNA repair and damage prevention in response to genotoxic stress. In this context, DMU-214 can be suggested to induce adaptive signals limiting its anti-cancer activity in A-2780 cells. All of the previous studies have focused only on a small group of apoptotic markers to evaluate apoptosis induction by DMU-212 and its metabolite DMU-214^6^,^10^,^11^,^15^. Hence, no broader cluster of genes involved in the cell death process has yet been identified. On the contrary, the microarray analysis applied in the present study allowed us to reveal expression changes of the other p53-target genes induced by DMU-214. We suggest that increased p48, p53R2, Gaad45 and sestrin mRNA levels, due to DMU-214 treatment, might be considered a mechanism of defence triggered in A-2780 ovarian cancer cells as a response to the insult evoked by the cytotoxic agent. Furthermore, the identified genes can be targets for developing new potential anti-cancer compounds. Regardless of the up-regulation of the above-mentioned genes involved in the cancer cells’ DNA repair, we demonstrated the stronger anti-proliferative and pro-apoptotic effects of DMU-214 in the A-2780 cell line when compared to those in SKOV-3. In view of these findings, we verified DMU-214 activity in the xenograft model using severely compromised immunodeficient (SCID) mice injected with A-2780 cells. We found that tumour growth in mice treated with DMU-214 was significantly lower as compared to untreated controls. These results are consistent with our previously published data showing the anti-proliferative activity of the parent compound DMU-212 in the xenograft model of human ovarian cancer^13^. Although the dose of DMU-214 was lower than that of the parent compound, we noted its stronger tumour inhibitory activity as compared to that of DMU-212. The substantial antiproliferative activity of DMU-214 in *in vivo* model allows us to suggest the tested compound as a potential therapeutic in human ovarian cancer treatment. Despite the potent anti-tumour effects of DMU-214, we observed no adverse effects during the entire period of the experiment.

In summary, our study demonstrated that DMU-214, the metabolite of DMU-212, displayed stronger anti-proliferative effects than the parent compound. Hence it was revealed, for the first time, that the DMU-212 cytotoxicity reported in previous experiments^10^,^11^,^13^ might be attributed to its metabolic activation. Furthermore, our results provide new insight into the role of DMU-214 in the modulation of the expression pattern of p53-target genes driving intrinsic and extrinsic apoptosis pathways as well as DNA repair and damage prevention in ovarian cancer cells with expression of wild-type p53. We found that in the cell line derived from the same type of tumour, but without p53, DMU-214 did not induce mitochondrial apoptosis. On the other hand, the tested compound triggered receptor-mediated apoptosis accompanied by strong cytotoxic activity. Hence we can suggest that the lack of the p53 protein did not abolish the anti-proliferative effects of DMU-214 in SKOV-3 cells, however, it did reduce its potency.

## Materials and Methods

### Chemicals and reagents

The metabolites of DMU-212 were synthesized as described elsewhere^6^. The identity and purity of each compound was confirmed by NMR and GC-mass spectroscopy. Propidium iodide (PI), ribonuclease A (RNase A), 3-(4,5-dimethylthiazol-2-yl)-2,5-diphenyltetrazolium bromide (MTT) and dimethyl sulfoxide (DMSO) were provided by Sigma-Aldrich Co. (St. Louis, MO, USA). The Cell Death Detection ELISA^PLUS^ kit was acquired from Roche Diagnostics (Germany). Caspase-Glo^®^ 3/7, Caspase-Glo^®^ 8 and Caspase-Glo^®^ 9 Assay Kits were purchased from Promega Co. (USA). The Affymetrix® Human Genome U219 Array Strip was provided by Affymetrix (Santa Clara, CA, USA). The Proteome Profiler Human Apoptosis Array kit was obtained from R&D Systems (Minneapolis, Minnesota, USA). Forane (*isoflurane*) was acquired from Abbott Laboratories Ltd. (Illinois, USA). D-Luciferin (potassium salt) was obtained from Gold Biotechnology (St. Louis, MO, USA).

### Animals

SCID female mice (CB17/Icr-Prkdcscid/IcrCrl), 6 weeks old and weighing 20–24 g, were obtained from Charles River Laboratories (Wilmington, MA, USA). The animals were housed (5/cage) in individually ventilated cages equipped with HEPA filters (Tecniplast, Italy). Room climate was maintained at a temperature of 22–23 °C and 40–65% relative humidity. A commercial diet (ISO 9001-certified laboratory feed, totally pathogen free, Altromin, Germany) and drinking water were available *ad libitum*. The experiment was conducted according to the guidelines of the European Directive 2010/63/EU for the care and use of laboratory animals. The experimental protocol was approved by the Local Animal Ethics Committee of Poznan University of Life Sciences (Poznan, Poland).

### Cell culture and cell viability assays

A-2780 and SKOV-3 ovarian cancer cell lines were purchased from the European Type Culture Collection (Sigma-Aldrich Co., St. Louis, MO). All cell lines were maintained in phenol red-free DMEM medium (Sigma-Aldrich Co., St. Louis, MO, USA), supplemented with 10% foetal bovine serum (FBS), 2 mM glutamine, penicillin (100 U/ml) and streptomycin (0.1 mg/ml) (Sigma-Aldrich Co., St. Louis, MO, USA). Cells were cultivated under standard conditions at 37 °C in a humidified atmosphere containing 5% CO_2_ and 95% air. To evaluate the effects of the four metabolites of DMU-212 on cell viability, confluent stock cultures were detached using trypsin and seeded in 96-well plates at a density of 2 × 10^4^ cells/ well in 100 μl of growth medium. They were allowed to attach overnight and the metabolite of DMU-212 was then added from the stock solution prepared in DMSO at a concentration of 100 mM/ml. The final concentration of DMSO in the cell treatment solutions was less than 0.1%. Control cells were cultured under the same conditions with 0.1% DMSO. Cell viability was measured spectrophotometrically using MTT as a substrate, as described elsewhere^32^. The concentration of DMU-214 that is required for 50% cell growth inhibition (IC_50_) was determined from a plot of percentage of cell viability *versus* logarithm of concentration.

### Assessment of apoptosis and necrosis induction

The Cell Death Detection ELISA^PLUS^ kit was used to detect apoptosis and necrosis in the A-2780 and SKOV-3 cell lines treated with DMU-214 according to the manufacturer’s instructions. Briefly, cells were seeded in a 96-well plate at a density of 2 × 10^4^ cells per well for 24 h, and DMU-214 at concentrations of 0.125 μM and 0. 250 μM was added. After 24 h, the lysate and supernatant were placed in a streptavidin-coated microtiter plate and incubated with a mixture of anti-histone-biotin, anti-DNA-peroxidase and incubation buffer. After 2 h of incubation, the unbound antibodies were removed by a washing step. Quantification of the nucleosomes was performed by measuring optical absorbance at 405 nm against the substrate solution as a blank (reference wavelength 492 nm).

### RNA extraction

Total RNA was extracted from the samples using TRI Reagent (Sigma, St. Louis, MO, USA) and the RNeasy Mini Kit (Qiagen, Hilden, Germany). The amount of total mRNA was determined from the optical density at 260 nm, and RNA purity was estimated using the 260/280 nm absorption ratio (higher than 1.8) (NanoDrop spectrophotometer, Thermo Scientific, ALAB, Poland). RNA integrity and quality were checked on the Bioanalyzer 2100 (Agilent Technologies, Inc., Santa Clara, CA, USA). The resulting RNA integrity numbers (RINs) were between 8.5 and 10 with an average of 9.2 (Agilent Technologies, Inc., Santa Clara, CA, USA). The RNA of each sample was diluted to a final working concentration of 100 ng/μl.

### Microarray expression analysis and statistics

All samples were prepared in triplicate. cDNA for the microarray analysis was synthesized using the Affymetrix GeneChip IVT Express Kit (Affymetrix, Santa Clara, CA, USA) according to the Affymetrix GeneAtlas protocol. Subsequent steps, i.e. transcription *in vitro*, biotin labelling and cDNA fragmentation, were also performed using the same protocol. Then the samples were loaded and hybridized with Affymterix GeneChip Human Genome U219 microarrays together with control cDNA and oligo B2. The hybridization process was conducted with the use of the AccuBlockTM Digital Dry Bath (Labnet International, Inc.) hybridization oven at 45 °C for 16 h. Then the microarrays were washed and stained according to the technical protocol using the Affymetrix GeneAtlas Fluidics Station. The array strips were scanned by the Imaging Station of GeneAtlas System. Preliminary analysis of the scanned chips was performed using Affymetrix GeneAtlasTM Operating Software. The quality of gene expression data was checked according to quality control criteria provided by the software. The obtained CEL files were imported into downstream data analysis software. All of the presented analyses and graphs were done by Bioconductor and R programming language. Each CEL file was merged with a description file. We used the Robust Multiarray Averaging (RMA) algorithm to correct the background and to normalize and summarize the results. Statistical significance of the analysed genes was performed by moderated t-statistics from the empirical Bayes method. The obtained p value was corrected for multiple comparisons using the Benjamini and Hochberg false discovery rate.

Differentially expressed genes involved in apoptosis were selected by using the DAVID database (Database for Annotation, Visualization and Integrated Discovery). Expression data of selected genes were subjected to hierarchical clusterization and presented as a heat map. The Pathview library of the bioconductor was used to generate the p53 signalling pathway. The expression fold is mapped by colours on native KEGG, p53 signalling pathway, where green represents up-regulated expression and red represents down-regulated expression levels in relation to the control cells.

### RT-qPCR

The expression levels of several selected genes were also validated with the aid of RT-qPCR (LightCycler*®* Instrument 480 MultiwellPlate 96, Roche, Mannheim, Germany). Using specific primers and probes, the LightCycler® 480 Probes Master kit was applied according to the manufacturer’s instructions. Specification of the reaction products was checked by determining the melting points (0.1 °C/s transition rate).

### Protein expression analysis

The Proteome Profiler Human Apoptosis Array kit (R&D Systems) was used following the manufacturer’s instructions. Briefly, equal amounts of proteins, isolated from the control and from DMU-214-treated A-2780 cells, were incubated with the apoptosis array for 24 h at 4 °C. After washing of the unbound proteins, a cocktail of apoptosis-detection HRP-conjugated antibodies was used to reveal the apoptosis-related proteins by chemiluminescence. The X-ray images were scanned and finally analysed using the ImageJ program.

The expression levels of several selected apoptosis-related proteins were confirmed using Western blot analysis. A total of 30 μg of protein was re-suspended in sample buffer and separated on 10% Tris-glycine gel using SDS-PAGE. Gel proteins were transferred to nitrocellulose, which was blocked with 5% milk in Tris-buffered saline/Tween. Immunodetection was performed with rabbit polyclonal anti-Bax Ab (sc-6236), rabbit polyclonal anti-Bcl-2 Ab (sc-492), rabbit monoclonal anti-p53 (S392) Ab (ab-33889), rabbit polyclonal anti-DR4 (sc-7863) and rabbit polyclonal anti-cIAP1(sc-7943) followed by incubation with goat anti-rabbit HRP-conjugated Ab (sc-2004). The membranes were also incubated with anti-actin HRP-conjugated Ab (sc-1616) to ensure equal protein loading of the lanes. Bands were revealed using SuperSignal West Femto maximum sensitivity substrate, Pierce Biotechnology Inc. (Rockford, IL).

### Determination of caspase-8, -9, -3/7 activity

A-2780 and SKOV-3 cells were seeded in a 96-well plate at a density of 2 × 10^4^ cells per well, and after 24 h they were treated with a vehicle (0.1% DMSO) or DMU-214 at 0.125 μM and 0.250 μM concentrations. Twenty-four hours later the medium was removed and caspase-8, −9 and −3/7 activity was determined using a luminescent Caspase-Glo^®^-8, -9, -3/7 assay kit (Promega, USA) according to the manufacturer’s protocol. Luminescence was measured using Tecan i-control (Mannedorf, Switzerland).

### Cell cycle analysis

For the cell cycle evaluation, the cells were fixed with ethanol. After 30 min incubation, the cells were re-suspended in 1 ml of phosphate buffered saline (PBS) containing 10 μg/ml propidium iodide and kept at 4 °C for 30 min prior to measurement. The stained cells were analysed in a FACSCanto flow cytometer (Becton-Dickinson). Data analysis and acquisition were performed using FACS Diva software (Becton-Dickinson).

### SCID mice xenograft model

Subcutaneous xenograft tumours derived from human ovarian cancer cell line A-2780 were generated in CB17-SCID mice. The A2780 cells were stably transfected with plasmid for luciferase using Lipofectamine LTX with Plus Reagent (Invitrogen, Carlsbad, CA, USA) and selected with Geneticin (Gibco, Eggenstein, Germany) at a concentration of 1000 μg/ml for 21 days. The luciferase reporter gene encoding plasmid pGL4.51 was purchased from Promega (Madison, WI, USA). Briefly, 2 × 10^6^ A-2780 cells in 0.1 mL PBS were subcutaneously inoculated in the left groin of SCID mice. At day 3 after injection, mice with a similar level of bioluminescence were divided into two groups (control and DMU-214-treated), with five animals each. Starting with the next day, the tested compound was administered subcutaneously at a dose of 40 mg/kg b.w. three times a week for three weeks. DMU-214 was first dissolved in ethanol and then mixed with PBS. The final concentration of ethanol in the mixture was 1%. The control group was given the vehicle. D-Luciferin was injected intraperitoneally at a dose of 150 mg/kg b.w., followed by inhaled isoflurane anaesthesia and bioluminescence imaging (IVIS 200, Caliper Life Sciences, Hopkinton, MA, USA). Clinical observations were performed on a daily basis and body weight, water and food intake were recorded. Images of tumour-bearing mice were captured three times a week and the data were analysed using the *Living Images* software package (Caliper Life Sciences). All animals were humanely sacrificed after 21 days of monitoring due to the excessive tumour burden.

### Statistical analysis

Data were expressed as means ± SD for three independent experiments. Statistical analysis was performed with one way analysis of variance ANOVA followed by a Student-Newman-Keuls test. P values less than 0.05 were considered statistically significant.

## Additional Information

**How to cite this article**: Piotrowska-Kempisty, H. *et al*. 3′-hydroxy-3,4,5,4′-tetramethoxystilbene, the metabolite of resveratrol analogue DMU-212, inhibits ovarian cancer cell growth *in vitro* and in a mice xenograft model. *Sci. Rep.* **6**, 32627; doi: 10.1038/srep32627 (2016).

## Figures and Tables

**Table 1:** Effect of the metabolites of DMU-212 on the viability of A-2780 and SKOV-3 ovarian cancer cell lines.

Cell lines tested	Time of exposure	Cell viability [%]			
		DMU-214	DMU-281	DMU-291	DMU-807
A-2780	24 h	25.39 ± 2.42***	94.55 ± 2.78	85.09 ± 7.91*	85.86 ± 4.51**
	48 h	13.69 ± 1.91***	83.42 ± 4.05**	83.97 ± 3.83**	77.79 ± 15.55*
	72 h	5.26 ± 0.7***	75.03 ± 11.86**	96.01 ± 6.75	89.06 ± 8.59*
SKOV-3	24 h	69.96 ± 7.49**	100.41 ± 8.49	86.90 ± 16.38	101.47 ± 3.24
	48 h	29.18 ± 1.49***	90.22 ± 1.25*	92.36 ± 1.95	97.04 ± 3.29
	72 h	19.86 ± 0.11***	78.34 ± 4.67**	93.16 ± 0.54	93.02 ± 6.14

The investigated cell lines were treated for 24 h, 48 h and 72 h with a vehicle or with the tested compounds at a concentration of 10 μM. Results of three independent replicates are presented as mean ± SD. ***P < 0.001, **P < 0.01 and *P < 0.05.
